# Prediction of clinical toxicity in locally advanced head and neck cancer patients by radio-induced apoptosis in peripheral blood lymphocytes (PBLs)

**DOI:** 10.1186/1748-717X-5-4

**Published:** 2010-01-28

**Authors:** Elisa Bordón, Luis Alberto Henríquez-Hernández, Pedro C Lara, Ana Ruíz, Beatriz Pinar, Carlos Rodríguez-Gallego, Marta Lloret

**Affiliations:** 1 Canary Institute for Cancer Research (ICIC), Las Palmas, Spain; 2 Clinic Sciences Department of Las Palmas de Gran Canaria University (ULPGC), Spain; 3 Radiation Oncology Department, Hospital Universitario de Gran Canaria Dr. Negrín, Spain; 4 Inmunology Department, Hospital Universitario de Gran Canaria Dr. Negrín, Spain

## Abstract

Head and neck cancer is treated mainly by surgery and radiotherapy. Normal tissue toxicity due to x-ray exposure is a limiting factor for treatment success. Many efforts have been employed to develop predictive tests applied to clinical practice. Determination of lymphocyte radio-sensitivity by radio-induced apoptosis arises as a possible method to predict tissue toxicity due to radiotherapy. The aim of the present study was to analyze radio-induced apoptosis of peripheral blood lymphocytes in head and neck cancer patients and to explore their role in predicting radiation induced toxicity. Seventy nine consecutive patients suffering from head and neck cancer, diagnosed and treated in our institution, were included in the study. Toxicity was evaluated using the Radiation Therapy Oncology Group scale. Peripheral blood lymphocytes were isolated and irradiated at 0, 1, 2 and 8 Gy during 24 hours. Apoptosis was measured by flow cytometry using annexin V/propidium iodide. Lymphocytes were marked with CD45 APC-conjugated monoclonal antibody. Radiation-induced apoptosis increased in order to radiation dose and fitted to a semi logarithmic model defined by two constants: α and β. α, as the origin of the curve in the Y axis determining the percentage of spontaneous cell death, and β, as the slope of the curve determining the percentage of cell death induced at a determined radiation dose, were obtained. β value was statistically associated to normal tissue toxicity in terms of severe xerostomia, as higher levels of apoptosis were observed in patients with low toxicity (p = 0.035; Exp(B) 0.224, I.C.95% (0.060-0.904)). These data agree with our previous results and suggest that it is possible to estimate the radiosensitivity of peripheral blood lymphocytes from patients determining the radiation induced apoptosis with annexin V/propidium iodide staining. β values observed define an individual radiosensitivity profile that could predict late toxicity due to radiotherapy in locally advanced head and neck cancer patients. Anyhow, prospective studies with different cancer types and higher number of patients are needed to validate these results.

## Findings

Interpatient heterogeneity in normal tissue reactions due to different treatments varies considerably [1]. Patients treated with radiotherapy (RT) will develop clinical toxicity and this may limit the success of the treatment [2]. The genetic and molecular mechanisms of therapeutic radiation sensitivity are still poorly understood [3,4]. The treatment of head and neck cancer includes surgery and, in advanced stages, radiation. Normal tissue toxicity induced by RT is the main limiting factor in the treatment progress. Knowledge of individual variations determining tolerance would be of great value. The ability of cells to detect and repair DNA damages will condition the intrinsic radiosensitivity [5]. The majority of radiosensitivity predictive factors are related to gene expression profiles [6,7], although other approaches have been recently proposed [8]. Flow cytometry evaluation of lymphocyte apoptosis has been established as a reliable method to measure radiation-induced damage [9]. Quantification of radiation-induced apoptosis (RIA) in peripheral blood lymphocytes (PBLs) has been proposed for the prediction of normal tissue responses after RT [10,11]. It has been published that radiation-induced T-lymphocyte apoptosis can significantly predict differences in late toxicity between individuals [12]. A correlation existed between low levels of RIA in lymphocytes and increased late toxicity after radiation therapy. Development of predictive assays for clinical implementation requires that the test employed displays both high reproducibility and low variation [13]. Intrinsic radiosensitivity is genetically determined and varies in dependence of the patient and the tumour type. The aim of the present study was to analyze radio-induced apoptosis of peripheral blood lymphocytes in head and neck cancer patients and explore their role in predicting radiation induced toxicity.

## Methods

Seventy nine consecutive patients with histological confirmed cell carcinoma of head and neck, diagnosed and treated in our institution and given inform consent, were included in the study. Apoptosis analyses were performed between November 2004 and July 2006. The study was approved by the Research and Ethics Committee of our institution. Mean age of patients was 55.81 ± 12.02 years (range 19-79, median 58). Clinic-pathological characteristics of patients are detailed in Table 1. Evaluation of clinical toxicity was made according to the Radiation Therapy Oncology Group (RTOG) acute and late morbidity scoring system that classifies toxicity of patients into different levels: grade 1 (mild) to 4 (severe). Clinical toxicity of patients was evaluated in each visit. The time point used corresponds to the last evaluation (Table 2). The mean follow-up was 37.02 ± 30.15 months (range 3-148, median 31). Treatment protocols varied in order to the stage of the disease and the general state of the patient (Table 1). Patients who were treated with conventional RT received 1.8-2 Gy per day to a total mean dose of 69.1 Gy (range 64.8-72.2). Patients who were treated with high-dose hyperfractionated RT received two daily fractions of 1.2 Gy separated by at least 6 hours to a total mean dose of 78.6 Gy (range 70.0-81.6). PBLs were isolated during follow-up from 10 ml of blood by density gradient centrifugation on Ficoll-Hypaque (Lymphoprep, Gybco) as previously reported [11]. The final concentration of cells was adjusted to 2 × 10^5 ^cells/ml in complete RPMI, and they were separated into four 25-cm^2 ^flasks. Cells were irradiated at room temperature with 1, 2 and 8 Gy, 6 mV × rays (Mevatron, Siemens, Germany) at a dose rate of 50 cGy/min. After irradiation, the preparations were incubated at 37°C in 5% CO_2 _during 24 hours. Post incubation, four samples of 1.5 × 10^5 ^cells from each flask (one negative control and three samples for triplicate study) were washed, centrifuged and incubated with 5 μl of monoclonal antibody CD45 APC-conjugated monoclonal antibody, permitting the exclusion of erythrocytes, debris, and leukocytes. The apoptosis analysis was determined by Annexin V kit (Pharmingen, Benton Dickinson) and propidium iodide (PI) as previuosly reported [11]. Flow cytometric analyses were performed on a FACScalibur flow cytometer (Benton Dickinson). Each sample was analyzed using 5000 events/sample acquired in list mode by a Macintosh Quadra 650 minicomputer (Apple Computer Inc., Cupertino). Data analysis was performed via three-step procedure using the Cellquest software (Benton Dickinson). Apoptosis levels were measured at four radiation doses (0, 2, 4, and 8 Gy) in triplicate. Statistical analyses were performed using the SPSS Statistical Package (version 15.0 for Windows) as previously reported [11].

**Table 1:** Characteristics of the patients in study (n = 79)

	Cases	Percentages
**Gender**		
Male	72	91
Female	7	9
**Cancer site**		
Oral cavity and Oropharynx	29	36.7
Larynx and Hypopharynx	26	32.9
Nasopharynx and Unknown origin/Multiple	24	30.4
**Stage**		
III	20	25.3
IVA	42	53.2
IVB	17	21.5
**Histology**		
Epidermoid	67	84.8
Others	12	15.2
**RT schedule**		
Conventional	35	44.3
Hyperfractionated	44	55.7
**Concomitant treatments**		
CMT	42	53.2
Surgery	20	25.3
Amifostine	23	29.1

RT: radiotherapy, CMT: chemotherapy

**Table 2:** Toxicity observed in 79 Head and Neck cancer patients

Late Toxicity	Grade 0	Grade 1	Grade 2	Grade 3
Cutaneous	26 (31.9%)	35 (44.3%)	17 (21.5%)	1 (1.3%)
Mucosa	44 (55.7%)	32 (40.5%)	2 (2.5%)	1 (1.3%)
Subcutaneous	33 (41.8%)	34 (43.0%)	11 (13.9%)	1 (1.3%)
Xerostomia	17 (21.6%)	28 (35.4%)	25 (31.6%)	9 (11.4%)
Larynx	54 (68.3%)	24 (30.4%)	1 (1.3%)	0 (0.0%)
Esophago	54 (68.3%)	18 (22.8%)	3 (3.8%)	4 (5.1%)

## Results

**Figure 1:**
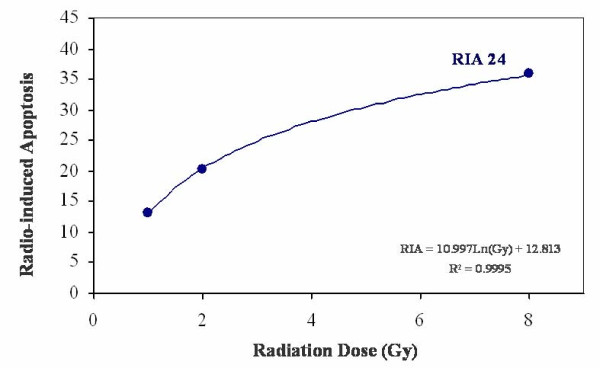
**Radio-induced apoptosis (RIA) of lymphocytes after 24 hours**. RIA values at 1, 2 and 8 Gy were adjusted perfectly to a semi logarithmic model defined by two constants: α is the origin of the curve in the Y axis and determines the percentage of spontaneous cell death and β is the slope of the curve and determines the percentage of cell death induced at a determined radiation dose.

Radio-induced apoptosis (RIA) could be defined as the percentage of total PBLs death induced by the radiation dose minus the spontaneous cell death (control, 0 Gy). RIA values increased with radiation dose (0, 1, 2 and 8 Gy) (Table 3), and fitted to a semi logarithmic equation as follow: RIA = β ln(Gy) + α (Figure 1). β values followed a normal distribution (mean 11.02 ± 3.61, range 4.02-19.61, median 11.32) and seems to represent a personalized marker of radiosensitivity. The adjustment coefficients (R) were determined and data strongly fitted to a semi logarithmic mathematical model. Correlation values at 24 hours were: mean 0.97 ± 0.44, median 0.99, range 0.76-1. Also, the intraindividual and interindividual variations were determined in the four healthy donors and in the 79 patients. Intraindividual variation for healthy donors was always lower than interindividual variation for patients (data not shown).

**Table 3:** Data of apoptosis and radio-induced apoptosis (RIA) of PBLs treated with 0, 1, 2 and 8 Gy of radiation at 24 hours.

Dose (Gy)	Apoptosis, 24 h	RIA, 24 h
0	39. 88 ± 14.80	
1	52.83 ± 13.30	13.00 ± 5.47
2	60.11 ± 11.97	20.15 ± 8.37
8	75.66 ± 10.53	35.78 ± 10.12

Cells were isolated from 79 Head and Neck cancer patients. Mean ± SD was included. RIA data followed a normal distribution (Kolmogorov-Smirnoff test, p = NS) and strongly fitted to a semi logarithmic model

RIA: Radio-induced apoptosis

**Figure 2:**
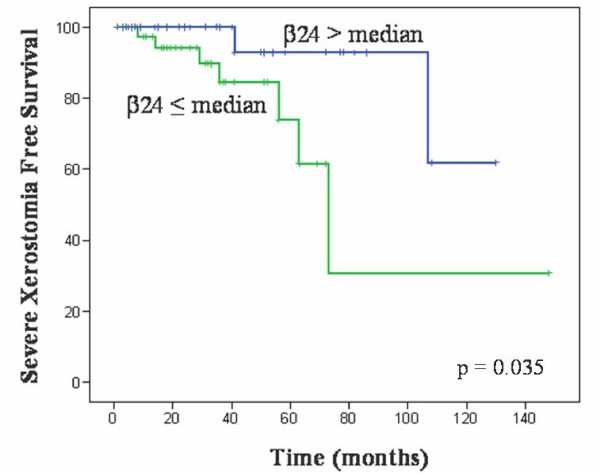
**Kaplan-Meier analysis of RIA values and development of severe xerostomia**. The analysis was made to establish a relationship between β radiosensitivity constant and the xerostomia free survival. Data were segregated based on the median distribution. Xerostomia in grade 3 was considered severe.

Cutaneous, mucosa, subcutaneous, laryngeal and esophageal toxicities as well as xerostomia were evaluated according to the RTOG scoring system (Table 2). The majority of patients did not suffer toxicity or suffered low grade of toxicity, especially mucosa (96.2%), laryngeal (98.7%) and oesophageal damage (91.1%). A Log Rank analysis was performed to evaluate the relationship between β and the different normal tissue toxicity reactions observed. Patients were segregated based on the median distribution of β value (cut-off ± 11.32). β 24 values below the median were related with higher severe xerostomia toxicity, grade 3 (p = 0.035; Exp(B) 0.224, I.C.95% (0.060-0.904)) (Table 4). As expected, toxicity was marginally associated with radiation schedule, that determines the total dose of radiation received (p = 0.058; Exp(B) 3.950, I.C.95% (0.955-13.88)) (Table 4). The Kaplan-Meier analysis makes visible the relation between β radiosensitivity constant and xerostomia grade 3 (Figure 2). Age at the time of diagnosis (patients were segregated according to the median age), gender, tumour localization, RT schedule and other concomitant treatments including chemotherapy, surgery and amifostine were analyzed as well. No association was observed in any case (Table 4).

**Table 4:** Relation between xerostomia free survival and different variables (Log Rank test)

Variables	Free survival at 60 months (%)	Exp(B), CI 95%; p value
**Age (years)**		
< 58	89.7	
>58	68.4	0.460 (0.109-1.702); 0.160
**Gender**		
Male	82.0	
Female	80.0	1.676 (0.261-10.22); 0.601
**Tumour localization**		
OC + Or ^a^	73.1	^(avs.b) ^2.166 (0.306-13.04); 0.524
L + H ^b^	100	^(avs.c) ^1.146 (0.281-4.712); 0.845
N + U/M ^c^	84.4	^(bvs.c) ^0.960 (0.101-9.101); 0.970
**RT schedule**		
Conventional	88.9	
Hyperfractionated	77.6	3.950 (0.955-13.88); 0.058
**CMT**		
Yes	87.2	
No	80	0.755 (0.188-2.926); 0.669
**Surgery**		
Yes	94.7	
No	76.8	3.910 (0.670-11.16); 0.161
**Amifostine**		
Yes	81.5	
No	85.5	0.617 (0.071-3.726); 0.510
**β 24**		
< 11.32	92.9	
> 11.32	73.9	0.224 (0.060-0.904); 0.035

OC: oral cavity, Or: oropharynx, L: larynx, H: hypopharynx; N: nasopharynx,

U/M: unknown origin/multiple, RT: radiotherapy, CMT: chemotherapy

## Discussion

Head and neck cancer is treated mainly by surgery and radiotherapy. Normal tissue toxicity due to radiotherapy (RT) limits the efficacy of the treatment. Different predictive toxicity assays have been developed [8]. Anyhow, analysis of radiation induced apoptosis (RIA) in peripheral blood lymphocytes (PBLs) by flow cytometry seems to be a useful approach to determine individual variability to RT [9]. We reported recently that RIA and late toxicity were related at different radiation doses and time points, and data strongly fitted to a semi logarithmic mathematical model defined by two constants: α and β [11]. In the present study we made the same approach in a set of 79 head and neck cancer patients. We observed that RIA values increased with radiation dose (0, 1, 2 and 8 Gy) and fitted to a semi logarithmic equation confirming our previously reports made in 94 cervix cancer patients. Higher levels of β values were significantly associated to lower levels of late toxicity. This finding agree with previous studies [9,14] where RIA presented higher levels in healthy patients compared with radio-sensitive patients and patients who suffered ataxia-telangiectasia (AT) [15] as well as in different subpopulations of lymphocytes [12,16]. The loss of salivary gland function is not life-threatening, but it can dramatically reduce the quality of life and may lead to impairment of social activities for long-term survivors [17]. Permanent mouth dryness can also result in sticky salvia, dental decay, and nutritional problems [17]. β value predicted only xerostomia in our study. This fact could be explained because xerostomia was the only toxicity reaction observed in a sufficient number of cases (43% of patients suffered grade 2-3 of xerostomia) as other severe toxicity reactions were infrequent even at higher radiation doses. Xerostomia was also associated with the total dose of radiation received. This finding agree with other studies where doses <26-30 Gy, using intensity-modulated radiotherapy (IMRT), significantly preserve salivary gland function [18]. In fact, xerostomia was predicted by β values at 24 hours. Moreover, in multivariate analysis β 24 was strongly associated with severe xerostomia with an Exp(B) of 1.583 (95% confidence interval, 1.075-2.331, p = 0.020). Amifostine is a cytoprotective agent against radiotherapy. The efficacy of amifostine has been a subject of clinical studies in different cancer types [19]. It has been reported that patients with head and neck squamous cell carcinoma treated with amifostine prior to RT had lower incidence of chronic xerostomia [20-22]. We did not observe this cytoprotective effect, probably due to the small number of patients who received amifostine (n = 23). Anyhow, amifostine was only approval for reduction of the incidence of xerostomia in patients undergoing postoperative RT alone for head and neck cancer. Despite this, the use of this agent remains limited [19]. PBLs apoptosis, measured as an integrated value of radio-sensitivity (from 1 to 8 Gy), seems to has the potential to predict which patients will be spared late toxicity after radiation therapy. Feasibility and cost effectiveness of this assay would favour larger studies to analyze the predictive role of this model, especially in different lymphocyte subpopulations. Anyhow, constant β, that defines the individual radio-sensitivity and represents the predictive value, need extensive and prospective studies to be validated.

## List of abbreviations

AT: Ataxia-Telangiectasia; PBLs: Peripheral Blood Lymphocytes; PI: Propidium Iodide; RIA: Radio-induced Apoptosis; RT: Radiotherapy.

## Competing interests

The authors declare that they have no competing interests.

## Authors' contributions

EB has made all the cell experiments with lymphocytes, irradiation of cells, flow cytometry experiments, data acquisition and statistical analyses. LAHH has written the manuscript and has been aware of the submission process. PCL has been involved in conception and design of the study and in drafting the manuscript and has given final approval of the version to be published.

AR, BP and MLl have made the selection of patients, the evaluation of clinical variables and grade of toxicity as well as all the aspects related with the patients selected, including the treatment. CRG has been involved in flow cytometry experiments as well as in RIA measurements. All authors read and approved the final manuscript.
